# Use of Nutritional Strategies, Bioactive Compounds, and Dietary Supplements in Young Athletes: From Evidence to Potential Risks—A Narrative Review

**DOI:** 10.3390/nu17132194

**Published:** 2025-06-30

**Authors:** Diego De Zan, Francesca Eletti, Giulia Fiore, Elisa Di Girolamo, Gaia Giulia Maria Bozzini, Veronica Perico, Martina Tosi, Lorenzo Norsa, Gianvincenzo Zuccotti, Elvira Verduci

**Affiliations:** 1 Department of Pediatrics, Vittore Buzzi Children’s Hospital, 20154 Milan, Italyelisa.digirolamo@unimi.it (E.D.G.); gaia.bozzini@unimi.it (G.G.M.B.); martina.tosi@unimi.it (M.T.);; 2 Department of Biomedical and Clinical Sciences, University of Milan, 20157 Milan, Italy; 3 Department of Health Sciences, University of Milan, 20142 Milan, Italy; 4 Metabolic Diseases Unit, Department of Pediatrics, Vittore Buzzi Children’s Hospital, University of Milan, 20154 Milan, Italy

**Keywords:** nutrition, young athletes, bioactive compounds, macronutrients, micronutrients, antioxidant vitamins, ergogenic supplements, nitrates, nutraceuticals

## Abstract

Young athletes face unique nutritional challenges due to their simultaneous engagement in intensive physical training and ongoing growth and development. Standard adult-based dietary recommendations often fail to meet the specific needs of this population. While the role of macronutrients and micronutrients is well recognized, increasing attention is being paid to bioactive compounds—non-essential food-derived elements with potential health benefits. This review aims to summarize current evidence regarding the efficacy, safety, and potential benefits of bioactive compounds in the nutritional management of young athletes. **Methods**: A narrative review of the literature published over the last 30 years was conducted across PubMed/Medline, Embase, and Scopus databases to identify relevant studies published in English. The inclusion criteria covered original research articles, clinical trials, cohort and case-control studies, and meta-analyses focusing on individuals aged 8–20 years. Studies addressing supplementation strategies, physiological effects, and safety concerns of bioactive compounds in young athletes were selected. Preclinical data and adult-based studies were also included to contextualize molecular mechanisms and support clinical findings. **Results**: The review highlights that bioactive compounds such as omega-3 fatty acids, curcumin, caffeine, and creatine as well as antioxidant vitamins may play a beneficial role in improving recovery, immune function, and performance in young athletes. Of these 21 studies, 8 focused on recovery and muscle soreness, 6 addressed immune function or antioxidant/anti-inflammatory effects, and 7 investigated direct performance enhancement. However, most of the available evidence derives from adult populations, and pediatric-specific data remain limited. Concerns remain about the misuse of supplements, lack of professional guidance, and potential contamination with banned substances. **Conclusions**: While some bioactive compounds show promising potential to support the health and performance of young athletes, current evidence is insufficient to support routine use in this population. More pediatric-specific research is necessary to establish safety, efficacy, and appropriate supplementation protocols tailored to young athletes’ unique physiological needs.

## 1. Introduction

Young athletes have specific nutritional needs due to the combined demands of growth, training, and competitions. In the absence of specific guidelines for pediatric athletes, adopting adult recommendations may seem practical but proves inadequate [1]. Active children and adolescents require balanced diets rich in vitamins, minerals (e.g., calcium, iron), proteins, and carbohydrates to support energy needs and bone health [2]. Internationally, the use of dietary supplements among young athletes is increasingly common, although data on prevalence and patterns of use vary across studies [3,4,5]. International surveys indicate that a significant proportion of adolescent athletes consume supplements, often without adequate professional guidance. These trends have been observed with varying rates depending on sport type, competitive level, and cultural context. Despite this, there is a lack of comprehensive data specifically focused on pediatric populations, highlighting the need for targeted research and reviews like the present one. This review aims to fill this gap by compiling and critically analyzing existing evidence on nutritional strategies and supplement use among young athletes, comparing pediatric data with adult populations, and identifying areas requiring further study.

Adolescence is marked by rapid growth, hormonal changes, and organ maturation, which influence body composition, metabolism, and nutrient requirements [6]. When athletic activity is added, particularly at competitive or elite levels, nutritional needs become even more specific and must be individualized according to sport, training intensity, age, and developmental stage [2,7,8]. To better distinguish between categories of physically active youth, McKinney et al. [9] proposed a classification of athletes based on training volume and competition level (Table 1). This distinction is useful to clarify that the present work focuses on young competitive and elite athletes, whose needs differ substantially from those of recreationally active peers [9,10].

Accurate estimation of the energy needs in young athletes is complex due to inter-individual metabolic differences. Inadequate energy intake may result in serious health consequences, including delayed puberty, menstrual dysfunction, compromised bone health, short stature, increased injury risk, and disordered eating behaviors [8]. Therefore, dietary planning must ensure sufficient energy intake, proper hydration, and a balanced intake of macronutrients and micronutrients [8].

In recent years, attention has turned toward bioactive compounds, non-nutrient substances in foods or supplements that may positively affect health beyond their basic nutritional role [11,12]. They include polyphenols (e.g., flavonoids), carotenoids (e.g., beta-carotene), omega-3 fatty acids, phytochemicals (e.g., glucosinolates), and dietary fiber [13]. Bioactive compounds are associated with various health benefits, including antioxidant activity, anti-inflammatory properties, improved immune function, and reduced risk of chronic diseases like cardiovascular disease or cancer [14]. However, all these potential benefits depend on the dosage and possible interactions with other components; despite these considerations, many athletes consume supplements containing bioactive compounds without adequate knowledge or professional guidance. Studies have highlighted a widespread lack of awareness regarding active ingredients, proper dosages, potential interactions and side effects, and the risk of contamination with banned substances.

This is particularly concerning among young people, as supplement use is often driven by peer influence or the belief that supplementation is essential for achieving success.

While some young athletes may rely on supplements to optimize performance or recovery, this does not equate to the deliberate or abusive use of bioactive compounds. Nonetheless, the distinction between informed use and misinformed or risky supplementation practices must be emphasized in educational strategies [15]. Studies indicate that, while the prevalence of doping among adolescent athletes remains relatively low, between 1% and 3%, according to recent surveys there is growing concern about its potential increase [16]. Furthermore, many young athletes consider the use of supplements as a normal and even necessary part of achieving sporting success, reflecting a widespread acceptance of performance-enhancing strategies. Notably concerning, there is also a common belief that other competitors may resort to banned substances in order to win, suggesting a progressive normalization of doping practices. These attitudes reveal a concerning lack of awareness among young athletes regarding the potential health risks and ethical implications associated with such behaviors [17]. However, athletes often lack adequate knowledge about the potential health risks associated with these substances [17]. Children and young athletes show distinct physiological responses to exercise compared to adults. For instance, prepubertal athletes rely more on heart rate than stroke volume to increase cardiac output [9], and their ventilatory responses during exercise are amplified, likely due to a lower CO_2_ threshold [10,11]. They rely more on lipid oxidation than adults in supporting endurance performance [12,13] and experience muscle development primarily through hypertrophy during puberty, which is more pronounced in males [15].

Given these assumptions, nutritional requirements in young athletes must not only optimize performance but also support healthy development and reduce future health risks. Research into the role of bioactive compounds in pediatric athletes is still limited and largely extrapolated from adult studies, which may not be directly transferable due to developmental differences. While some ergogenic aids may hold promise, their use in youth must be carefully evaluated under medical supervision. This review aims to summarize current evidence on the nutritional needs of children and adolescent athletes and critically assesses the role and safety of bioactive compounds and dietary supplements in the same population.

## 2. Materials and Methods

This literature review was performed by a comprehensive search of the literature published in the last 30 years (from 1994 to 2024), using the following electronic databases: Pubmed/Medline, Embase, and Scopus. The aim of the search was to identify studies examining the potential impact of bioactive compounds on athletic performance and overall health in young athletes (8–20 years). Inclusion criteria were (i) original research articles, randomized or non-randomized clinical trials, cohort or case-control studies, and meta-analyses; (ii) studies involving pediatric or adolescent athletic populations (8–20 years); and (iii) interventions evaluating the use of bioactive compounds or dietary supplements in relation to health, recovery, or performance. Exclusion criteria were (i) narrative reviews, editorials, and opinion papers; (ii) studies involving only adult populations (unless used for contextual purposes); and (iii) animal or preclinical studies not directly supporting clinical findings. Preclinical studies were considered only to complement descriptions of bioactive compounds mechanisms of action, thereby unifying the molecular basis with the evidence from clinical trials.

The search was performed considering all articles published on this topic written exclusively in English, by means of the following keywords (alone or in combination): “nutrition”, “young athletes”, “bioactive compounds”, “macronutrients”, “micronutrients”, “protein”, “omega 3”, “EPA”, “DHA”, “vitamin D”, “vitamin A”, “vitamin C”, “antioxidant vitamins”, “iron”, “calcium”, “fluids”, “curcumin”, “caffeine”, “creatin”, “ergogenic supplements”, “nitrates”, and “nutraceuticals”. Article selection was performed by two independent reviewers who screened titles and abstracts based on predefined inclusion criteria. Reference lists of these articles were also examined to identify additional relevant studies. Data extracted from the selected studies were synthesized using a narrative approach, organizing evidence around key aspects including the potential benefit of the bioactive compound under study, reference age of athletes, and dosage and duration of administration. There are 21 articles included in the final review, with the following study designs: 6 clinical trials, 12 RCTs, and 3 systematic reviews and meta-analyses.

After a first overview of the current evidence regarding the targeted nutritional requirements of young athletes, the results on bioactive compounds are presented according to three main categories: micronutrients of key interest for prevention, well-being and performance (e.g., vitamin D, Calcium, and Iron); supplements that may assist with training capacity, recovery, muscle soreness, and injury (antioxidants such as omega-3, vitamin C, and Curcumin); and ergogenic supplements (e.g., Creatin, Caffeine, Nitrate, and Beta-Alanine). This classification is inspired by and based on those developed for the adult population by the IOC and the American College of Sports Medicine, particularly considering the two consensus statements: Dietary Supplements and the High-Performance Athlete [7] and Nutrition and Athletic Performance [18]. A flow chart summarizing the article selection process is provided in the Supplementary Materials.

## 3. Results

### 3.1. Targeted Nutritional Requirements in Young Athletes

Young athletes exhibit higher energy requirements compared to non-athlete peers due to simultaneous energy expenditure for growth and sport performance [19,20]. Currently, there are no predictive equations capable of accurately determining the energy needs of adolescent athletes: markers of growth and development should be compared with reference standards throughout the adolescent athlete’s life to determine if total energy intake is adequate [6]. Since specific nutritional guidelines for pediatric athletes are currently lacking, it becomes necessary to adapt recommendations developed for adults. The Sports Dietitians Australia Position Statement suggests that, in the absence of specific evidence for adolescent athletes, the most prudent approach is to follow the guidelines for adult populations regarding the consumption of proteins, carbohydrates, and fats. In fact, there is no evidence to suggest that the carbohydrate requirements of adolescents differ significantly from those of adults, so adolescent athletes should adjust their carbohydrate intake according to their actual daily energy needs, following the same recommendations as for adults. Similarly, the consumption of fats should align with public health guidelines, prioritizing unsaturated fats and limiting saturated fats, in line with recommendations for the general population [6]. The Dietary Reference Values issued by the European Food Safety Authority (EFSA) are a pivotal reference for this population [21].

An overview of current evidence-based advice for macronutrient distribution, key micronutrients, and hydration strategies for young athletes, adapted from adult recommendations, is summarized in Table 2.

### 3.2. Bioactive Compounds in Young Athletes

A total of 423 articles were initially retrieved through database searching. After removing duplicates (n = 76), 347 articles were screened by title and abstract. Of these, 285 were excluded for not meeting the inclusion criteria. Sixty-two full-text articles were assessed, and 41 were excluded due to reasons such as adult-only populations, lack of outcome data, or narrative/review design.

The final 21 studies included in the narrative synthesis focused specifically on bioactive compound supplementation in youth athletes (aged 8–20 years) and consisted of 6 clinical trials, 12 randomized controlled trials, and 3 systematic reviews/meta-analyses.

Of these 21 studies: 8 focused on recovery and muscle soreness, 6 addressed immune function or antioxidant/anti-inflammatory effects, and 7 investigated direct performance enhancement (e.g., creatine, caffeine).

The International Olympic Committee (IOC) consensus statement highlights that, while dietary supplements are widely used among athletes for various reasons, only a limited number have robust evidence supporting their efficacy for performance or health. The use of supplements should be based on a thorough nutritional assessment and guided by expert advice, as inappropriate use can pose health risks or result in inadvertent doping violations. Most evidence-based recommendations concern supplements such as omega-3, vitamin C, and Curcumin, as well as ergogenic supplements like caffeine, creatine, beet-nitrate, and beta-alanine, while the benefits of many other supplements remain unproven [7]. The list of bioactive compounds studied to optimize athletic performance and their current evidence in the pediatric population is presented in the following sections and summarized in Table 3.

#### Micronutrients of Key Interest for Prevention, Well-Being, and Performance

Supplementation with vitamin D, calcium, and iron is therefore considered essential in young athletes, not primarily for performance enhancement but to ensure the maintenance of optimal micronutrient status, as deficiencies in these elements can lead to significant health consequences. Adequate intake of calcium and vitamin D is crucial for young athletes to maximize peak bone mass and reduce the risk of fractures and osteoporosis [60]. Pediatric athletes, especially those who train indoors during the winter season, are at a higher risk of vitamin D deficiency, which has significant implications for achieving optimal peak bone mineral density (BMD) during adolescence.

For this reason, correction of any vitamin D deficiency or insufficiency through supplementation may be necessary to ensure optimal performance and bone health in adolescent athletes [6]; however, when adequate vitamin D status is present, its supplementation to further increase serum 25(OH)D concentrations does not seem to confer additional benefits to muscle strength, power, and physical function [61]. A single mega-dose of vitamin D3 (200.000 UI equivalent to 5000 µg) in vitamin D-deficient young amateur soccer players has been shown, in a randomized controlled trial [24], to improve jumping ability, agility, and running speed. In contrast, evaluating the effect of 2000 UI/day (50 µg/day) of vitamin D_3_ supplementation for 12 weeks on physical performance in adolescent swimmers with insufficiency did not reveal a significant difference between those who became sufficient and those who did not [25]. The authors of this study suggest that further intervention studies are warranted in different sports disciplines, using higher doses and longer durations of vitamin D supplementation, before final conclusions on this topic.

Calcium supplementation of 21.5 mg/kg body mass 60 min before the physical tests also proved to be an ineffective strategy for enhancing anaerobic and neuromuscular performance, as demonstrated in a study involving fifteen soccer players under the age of 15 [26].

Iron is another essential micronutrient for young athletes, as its deficiency is linked to reduced physical performance due to its role in oxygen transport to tissues and its involvement in energy metabolism. Iron supplementation may positively influence physical performance in children, with significant benefits with respect to the blood lactate levels and endurance time [27]. A 2014 systematic review and meta-analysis assessed the effect of iron supplementation compared to controls on physical performance in women of reproductive age (12–50 years) [28]. It was demonstrated that iron supplementation (from 1 mg/day to 325 mg/day of elemental iron, for 4–24 weeks) improved both maximal exercise performance, as indicated by an increase in maximal oxygen consumption (VO_2_ max), and submaximal exercise performance, as evidenced by a lower heart rate and a reduced proportion of VO_2_ max required to achieve defined workloads. This was the first study to provide systematic evidence on the effects of iron supplementation on athletic performance in women of reproductive age, including adolescents. However, as noted by the authors, there are several limitations, most notably the poor methodological quality of most of the interventions included. Nevertheless, the biological plausibility of supplementation remains strong, and there is a clear need for further research to confirm these findings on a broader scale. It is also important to emphasize that the Sports Dietitians Australia (SDA) states that iron supplementation should be considered only if medically indicated [6]. In line with this, we underline that the bioavailability of dietary iron rather than just the total iron content plays a critical role in determining iron status. Therefore, improving iron absorption through dietary strategies (e.g., pairing iron-rich foods with vitamin C sources and avoiding inhibitors like phytates) may be more effective than merely increasing total iron intake [62].

Inadequate energy intake is not only associated with impaired growth and performance but often leads to insufficient intake of key micronutrients such as iron, calcium, and vitamin D. Therefore, energy availability is a crucial determinant of overall nutritional status in adolescent athletes. Deficiencies in these micronutrients can result in serious health consequences such as the Female Athlete Triad, a medical condition commonly observed in physically active girls. The triad is characterized by at least one of the following components: low energy availability (EA), with or without disordered eating (DE), menstrual dysfunction, and low bone mineral density (BMD). Early intervention is critical to prevent progression to serious health issues, including clinical eating disorders, amenorrhea, and osteoporosis [63]. Recent research has also shown that low energy availability can impact many physiological functions beyond the three components of the triad. For this reason, the IOC Consensus group introduced a more comprehensive term, Relative Energy Deficiency in Sport (RED-S) [64]. The core issue of RED-S is insufficient energy intake to sustain the various bodily functions necessary for optimal health and performance, a situation that may be particularly challenging for growing adolescents. Low energy availability has multiple health and performance consequences, including menstrual cycle disruptions and increased risk of bone stress injuries; it may contribute to iron deficiency and impact thyroid function, fertility, and even psychological well-being [65]. Energy deficiency in athletes is also associated with cardiovascular consequences, such as early atherosclerosis, gastrointestinal symptoms, immunological alterations, psychological problems, and decreased athletic performance. Early detection of athletes at risk is essential to prevent the syndrome, particularly in young athletes, as 90% of peak bone mass is reached by 18 years of age, offering a critical window to optimize bone health [66]. The primary goal of treatment is to restore or normalize body weight, along with an improvement in overall nutritional and energetic status: this is considered the most effective strategy for resuming menstruation and improving bone health [63].

### 3.3. Supplements That May Assist with Training Capacity, Recovery, Muscle Soreness, and Injury

#### 3.3.1. Omega-3

##### Effects of Omega-3 Fatty Acids

Omega-3 fatty acids are essential polyunsaturated fats crucial for brain development, immune function, and cardiovascular health. Interest in omega-3 fatty acid supplementation for young athletes stems from evidence in adults, where fish oil or purified EPA and DHA extracts have shown positive effects on well-being and performance [67,68]. These benefits are linked to increased cellular membrane fluidity, improved muscle protein synthesis, and reduced inflammation for enhance recovery. However, physical activity also improves erythrocyte function [69], and studies do not consistently support a direct impact of omega-3s on maximal oxygen consumption (VO_2_ max) or performance [30,31]. In young athletes, specific evidence is limited, with most data extrapolated from adult studies.

##### Mechanisms and Interactions

In adults, 6 g of fish oil supplementation for six weeks has been shown to increase the omega-3 content in erythrocyte membranes, improving cellular fluidity and potentially enhancing oxygen uptake in muscles during exercise [29]. Adequate omega-3 intake may be beneficial in young, injured athletes to mitigate muscle loss during periods of inactivity [70,71]. In adults, 4 g omega-3 supplementation a day for 8 weeks (1.86 g EPA and 1.5 g DHA) enhances muscle protein synthesis (MPS) by increasing amino acid and insulin-mediated responses [32,33], which may help counteract muscle atrophy during recovery [72]. However, data on adolescent athletes are lacking, and the optimal omega-3 dosage for this age group remains unclear [73]. As for inflammation, omega-3 regulate eicosanoid synthesis and may mediate exercise-induced inflammation, aiding recovery. Intense physical exercise, especially with eccentric loads, can lead to exercise-induced muscle damage (EIMD), leading to delayed-onset muscle soreness (DOMS) [74]. Studies in adults suggest that around 1–2 g of omega-3s per day can reduce post-exercise inflammation and DOMS, indirectly benefiting performance [7,75]. However, no studies have examined these effects on adolescent athletes. Given the overall health benefits of omega-3s, obtaining adequate intake through diet, such as consuming two servings of fatty fish per week, is recommended. Supplementation is recommended for those who do not consume fish regularly.

##### Recommendations and Conclusions

Given their health benefits and anti-inflammatory properties, omega-3s can be considered a supportive component in the diet of adolescent athletes, especially during recovery phases. A practical recommendation is to encourage consumption of two servings of fatty fish per week. Supplementation may be considered in individuals with low fish intake, but routine use in all adolescents is not supported due lack of pediatric-specific evidence. More research is needed to clarify their role in sports performance in youth [7].

#### 3.3.2. Vitamin C

##### Effect of Vitamin C

Vitamin C is a potent antioxidant that not only acts as a scavenger but also converts vitamin E into its active form and enhances the absorption of iron from dietary sources. However, evidence regarding the effectiveness of vitamin C supplementation in athletes remains conflicting. Some studies found no significant changes in plasma ascorbic acid levels or reduction in oxidative stress following supplementation, while others reported increased erythrocyte glutathione levels, suggesting antioxidant activity [34,35]. Other beneficial effects of vitamin C supplementation have been reported in the literature. For instance, Constantini et al. observed that administration of 1 g/day of vitamin C in male adolescent swimmers resulted in halving the duration of upper respiratory tract infections and reducing their severity [36]. Some scientific evidence suggests that oxidative stress induced by intense physical exercise may impair athletic performance. Although vitamins C and E are potent antioxidants, there is no conclusive evidence demonstrating a positive effect on physical performance in individuals supplemented with these two vitamins [39,40].

##### Interactions with Other Components

The effects of vitamin C may also be enhanced by the concurrent intake of other vitamins and trace elements. Żychowska et al. demonstrated that administering vitamins A (16 µg/kg/day), C (8 mg/kg/day), and E (1 mg/kg/day) to adolescent athletes reduced the effects of oxidative stress induced by intense physical exercise. This reduction was associated with decreased gene expression of HSPA1A and HSPB1 (heat shock proteins, which are key mediators of cellular stress) in leukocytes [38]. Some researchers have hypothesized a potential pro-oxidant effect of vitamin C, suggesting it may contribute to the conversion of Fe^3+^ to Fe^2+^, which could lead to the formation of reactive oxygen species (ROS). This hypothesis assumes that physical exercise causes cellular damage, releasing the intracellular contents. However, no pro-oxidant effects of vitamin C have been observed in vivo so far [76].

##### Recommendations and Conclusions

The body’s response to supplementation, however, depends on factors such as dose, duration of administration, and initial plasma concentrations of ascorbic acid. Athletes with low circulating levels of ascorbic acid may show more significant responses to supplementation: several studies have shown that a 500 mg intake over a few days can lead to a 40–50% increase in vitamin C concentrations [37]. However, some studies suggest that there may be an initial transient increase following supplementation, followed by a decline with long-term administration. Levine et al. reported that an intake of 60 mg of vitamin C for three weeks leads to plasma saturation and the maximum cellular capacity for vitamin C [34,77]. While vitamin C may be useful to correct deficiencies or improve recovery in specific contexts, routine high-dose supplementation is not recommended in healthy adolescent athletes due to the lack of consistent performance benefits and the possible interference with training adaptations [7,78,79,80].

#### 3.3.3. Curcumin

##### Effects of Curcumin

Curcumin, the active compound in turmeric [81], has antioxidant and anti-inflammatory properties that may reduce delayed onset muscle soreness (DOMS) and improve recovery after intense exercise [42]. These effects are particularly relevant for athletes undergoing unaccustomed or eccentric exercise, which induces exercise-induced muscle damage (EIMD), inflammation, and oxidative stress [74,82]. A study in middle and high school athletes engaged in wrestling, soccer, and soft tennis (21 men and 7 women, with a mean age of 17 years) reported that 1200 mg/day of curcumin for 12 weeks reduced muscle pain and fatigue, but methodological limitations prevent definitive conclusions [44]. 

##### Mechanisms and Interactions

Curcumin’s high-bioavailability formulations enhance its low natural absorption, often by using enhancers like piperine [83]. Curcumin modulates oxidative stress by increasing antioxidant enzyme activity (SOD, GSH, catalase) and reducing lipid peroxidation; it also inhibits NF-kB activation, limiting pro-inflammatory cytokine expression [41], which may mitigate exercise-induced inflammation and promote recovery [42]. These effects would mitigate the inflammation and oxidative stress induced by exercise, limiting DOMS and promoting better athletic performance and recovery, and indirectly lowering the risk of injury [42]. However, chronic high-dose antioxidant supplementation (such as vitamin E and C and curcumin) could blunt training adaptations by attenuating the necessary oxidative and inflammatory responses to exercise [84].

##### Recommendations and Conclusions

The EFSA establishes the acceptable daily intake for adults at 3 mg/kg/day, which is the amount that can be safely consumed daily throughout life without recognizable adverse effects, according to the Italian National Institute of Health [85]. In the pediatric population, the administration of curcumin at doses ranging from 45 mg to 4000 mg/day for 2–48 weeks has been found to be safe and well tolerated [43]. Curcumin supplementation may be beneficial for young athletes undergoing intense or frequent training sessions, particularly in cases of multiple daily workouts or closely spaced competitions [80]. More research is needed to determine effective dosages, bioavailability, and long-term impact on performance [45].

### 3.4. Ergogenic Supplements

#### 3.4.1. Creatine

##### Effects of Creatine

Creatine supports energy production in high-intensity exercise by increasing phosphocreatine stores in muscles, enabling rapid ATP regeneration. Studies on adults’ supplementation have shown an increase in intramuscular phosphocreatine by 15–40%, enhancing sports performance and training adaptations. A physiological and metabolic rationale supports its use in adolescent populations, particularly in anaerobic sports, due to its ability to improve sprint capacity, muscular strength, power output, sport-specific skills, and repeated high-intensity efforts. Studies in young athletes (ages 14.3–20), quantitatively very limited compared to the number of interventions conducted in adults, primarily examined swimmers and soccer players, with no adverse effects observed [46,47,48,49,50,51,52,86].

##### Mechanisms and Interactions

The international society of sports nutrition (ISSN) identifies creatine monohydrate as the most effective form. Most studies involved a loading phase (20–30 g/day for 4–9 days) followed by a maintenance dose (5 g/day). Almost all interventions showed performance improvements such as sprint ability, muscular strength, power output, sport-specific skills, and repeated high-intensity efforts, with no reported side effects [46,47,48,49,50,51,52]. Supplementing with high doses of creatine monohydrate (e.g., 0.3 g/kg/day for 5–7 days or 20 g/day for 5 days) is indeed the most effective strategy to rapidly increase intramuscular creatine levels, as recommended by the International Society of Sports Nutrition (ISSN) [87] and the International Olympic Committee (IOC) [7]. Subsequently, to maintain elevated creatine stores, the most effective strategy is to continue with a daily intake of 0.05 to 0.15 g/kg or 3–5 g per day [7,87]. However, as evidenced by studies in adults, a loading phase is not necessary unless rapid saturation of intramuscular creatine stores is desired. For example, if an athlete is hoping to maximize the ergogenic potential of creatine supplementation in a very short period of time (<30 days), a ‘loading phase’ could be a recommended option [88]. On the other hand, if an athlete is planning to ingest creatine over an extended period of time (>30 days), current recommendations suggest consuming approximately 3–5 g/day of creatine for a minimum of 4 weeks to achieve similar levels of skeletal muscle saturation [88].

##### Recommendations and Conclusions

Many young athletes already use creatine independently [89,90], though evidence regarding its long-term safety in adolescents remains limited [86]. Most available safety data are self-reported, and studies often lack comprehensive laboratory assessments [88,91]. Nevertheless, the ISSN, based on the limited evidence, defines creatine supplementation as being safe and potentially beneficial for children and adolescents [88]. Considering the available evidence is not sufficient to approve its use in young athletes, there is a need for scientifically controlled studies aimed at determining the safety of creatine use in adolescents.

#### 3.4.2. Caffeine

##### Effects of Caffeine

Caffeine is the most widely consumed psychoactive substance, naturally found in coffee, tea, and cocoa. Its consumption is increasing among young adults and athletes through energy drinks, pre-workout supplements, chewing gum, and gels [92]. In adults, caffeine is a well-studied ergogenic aid, enhancing endurance, repeated sprint performance, and neuromuscular function [7]. While its benefits in endurance sports are well established, its effects on muscle endurance, strength, and power remain debated [92]. Other factors influencing its ergogenic effects include sex, intake timing, genotype, habitual use, and training status [93].

##### Mechanisms and Interactions

Caffeine acts primarily by antagonizing adenosine receptors, increasing neurotransmitter release, and reducing the perception of effort during exercise. It may also enhance glycogen resynthesis when combined with carbohydrates post-exercise [53]. The EFSA states that single caffeine doses up to 3 mg/kg body weight are safe for adults, a guideline also applied to children and adolescents [94]. Regular intake up to approximately 3 mg/kg per day does not seem to induce behavioral changes in youth, though 1.4 mg/kg doses may increase sleep latency and reduce sleep duration, especially if consumed near bedtime [95]. Genetic variability and sex may be key factors in determining caffeine’s safety in children. Research on caffeine’s impact on sport performance in youth is limited and mostly focused on males. A 2024 study found that acute supplementation of 6 mg/kg caffeine, taken 1 h before the Wingate test, improved anaerobic performance in highly trained male futsal players (15.9 ± 1.2 years) [54], supporting its potential benefits for strength and power activities [92]. Caffeine’s ergogenic effects may be stronger in trained individuals due to higher density of A2A adenosine receptors in skeletal muscle [54]. Other interventions confirm that acute supplementation of 3–6 mg/kg caffeine in capsule form enhances anaerobic performance in elite adolescent athletes [55,56,57], particularly in outcomes such as reaction times, agility and sprint speed, jump height, and post-exercise recovery. It is interesting to note that the intervention by Ellis M. et al. [57] showed that low doses (1–3 mg/kg) already have ergogenic effects, although these are task dependent. Moreover, supplementation with 3 mg/kg led to performance improvements across the majority of tests, suggesting that it could be an effective yet safe dose, especially for adolescents.

##### Recommendations and Conclusions

Caffeine may be considered a potentially safe and effective ergogenic aid in adolescents when used responsibly, particularly in strength and anaerobic sports. Based on its primary mechanisms of action and the evidence in adults, caffeine supplementation, whether in the form of drinks, capsules, gels, or gum, could potentially be ergogenic and hypothetically safe in aerobic performance for adolescents, although no interventions have yet been conducted in these populations. It is also important to consider individual sensitivity to side effects such as restlessness, anxiety, insomnia, tachycardia, and gastrointestinal discomfort [96], which were not reported in intervention studies on adolescents (although sample sizes were limited). Side effects could have a significantly negative impact on performance, so individualization and monitoring of supplementation for this age group are of primary importance. Further studies are needed to assess long-term safety, gender differences, and genetic variability in response to caffeine.

#### 3.4.3. Beet Nitrates

Dietary nitrates (NO3-) are studied for their potential benefits in submaximal and high-intensity exercise. Their main mechanism involves increased nitric oxide (NO) bioavailability, enhancing blood flow to skeletal muscles, improving type II muscle fiber function, and making mitochondrial respiration more efficient, reducing ATP consumption [7].

Rich dietary sources include leafy greens and root vegetables like spinach, lettuce, and beetroot [97]. Beetroot, in particular, is widely studied and consumed as powder or juice. The Australian Institute of Sport (AIS) and the IOC recognize its potential ergogenic effects [7,98]. However, systematic reviews highlight the limited number of studies and their heterogeneity in participant level, exercise protocols, dosages, and supplementation strategies [58,99]. Effects in females appear insignificant, likely due to methodological inconsistencies [100]. In adolescents, findings are inconclusive. A study on male basketball players (age 15.6 ± 0.5 years) found no significant performance improvements after acute beetroot juice ingestion (3 h prior) [101]. Similarly, a two-week intervention in soccer players (16–19 years) showed no VO_2_ max benefits compared to a control group. The mean ΔVO_2_ max was 3.13 ± 2.76 mL/kg/min in the beetroot juice group versus 4.43 ± 3.47 mL/kg/min in the control group, with no statistically significant difference between the groups (*p* = 0.42) [102]. Researchers suggested that inadequate energy intake might limit supplementation effects. Given its biological plausibility, further studies in young athletes are needed.

##### 3.4.4. β-Alanine

β-alanine is the limiting precursor of carnosine (β-alanylal-histidine), a key intracellular buffer that mitigates acidosis during muscle contraction [103]. In adults, supplementation offers small but potentially meaningful performance gains (~0.2–3%) in continuous and intermittent efforts lasting 30 s to 10 min [7]. The ISSN highlights its benefit in anaerobic activities of 2–4 min, where acidosis is a limiting factor [104]. Carnosine also has antioxidant properties and may influence calcium kinetics in muscle fibers, though these effects remain unconfirmed in humans [104].

Evidence in adolescents is limited. A study on water polo athletes (16 ± 2 years) found that 4.8 g/day for 5–6 weeks improved high-intensity performance, aligning with adult data. However, post-supplementation muscle carnosine levels were not measured, leaving uncertainty about accumulation in post-pubertal adolescents [59].

Paresthesia (i.e., tingling) is the most recognized side effect, but there is no evidence of health risks [104]. β-alanine is generally considered safe [104], though its use in adolescents requires caution. It may be beneficial in elite young athletes under medical supervision, but paresthesia could negatively impact performance by causing discomfort [104].

## 4. Discussion

**Figure 1:**
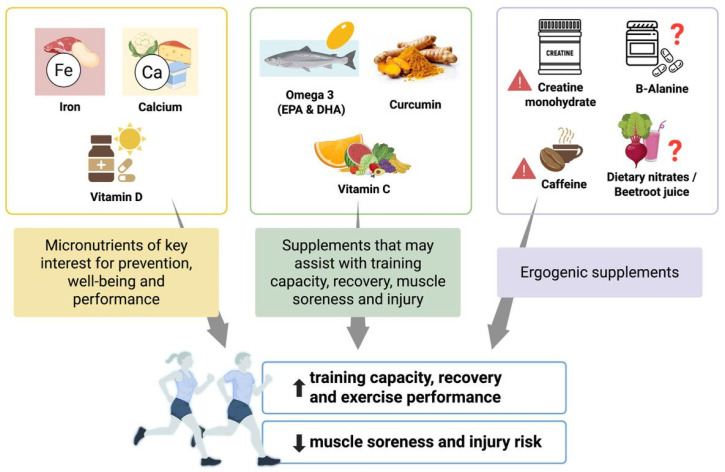
Potential benefits of bioactive compounds. A warning symbol has been added for those bioactive compounds with limited or inconclusive evidence in young athletes.

The use of bioactive compounds and dietary supplements among adolescent athletes is a growing phenomenon, driven by the desire to optimize performance, recovery, and overall athletic development [3,4,5,15]. Despite the increasing popularity of ergogenic aids in this age group, the available scientific literature remains limited and, in many cases, inconclusive. As shown in Figure 1, dietary supplements can be broadly categorized into micronutrients essential for health, compounds that may support training capacity and recovery, and ergogenic aids with performance enhancing potential.

Caffeine is one of the most studied ergogenic substances in adults, with well-documented effects on endurance, repeated sprint ability, and neuromuscular function through mechanisms such as adenosine receptor antagonism, increased endorphin release, and enhanced alertness [7]. In adolescents, the research is limited but promising. Acute supplementation with caffeine (3–6 mg/kg) has shown improvements in anaerobic performance markers such as reaction time, agility, and sprint speed in elite adolescent athletes [52,53,54]. One study highlighted the efficacy of lower doses (1–3 mg/kg), although the effects appeared to be task dependent [57]. However, caffeine’s impact on muscle strength and power remains debated even in adults [92]. Importantly, no side effects such as anxiety or gastrointestinal discomfort were reported in the adolescent studies, but limited sample sizes warrant caution [96]. Genetic variability, habitual use, sex differences, and timing of intake all represent factors that may modulate caffeine’s efficacy and safety in youth populations [92,93,94]. Given its established mechanisms and acute benefits, caffeine could be considered a potentially safe and effective ergogenic aid in adolescents, provided that individual responses are carefully monitored and low to moderate doses are used [94].

Dietary nitrates, particularly from beetroot juice, are recognized for their potential to enhance muscle efficiency and blood flow via increased nitric oxide availability [7]. While ergogenic effects have been observed in adults, especially in submaximal and high-intensity exercise, the evidence in adolescents remains limited and inconclusive. Studies in male basketball and soccer players did not show significant improvements in performance following acute or short-term beetroot supplementation [101,102]. Factors such as inadequate energy intake, sex-related physiological differences, and protocol variability may have influenced these outcomes [99,100]. Given the biological plausibility and positive trends in adult populations, more controlled investigations are needed to determine the effectiveness of beetroot-derived nitrates in youth athletes.

β-Alanine, a precursor of carnosine, acts as an intracellular buffer to counteract acidosis during high-intensity efforts. In adults, supplementation has been associated with small but meaningful improvements in exercise performance lasting from 30 s to 10 min [7,104]. Preliminary data in adolescent water polo players showed promising results after 5–6 weeks of supplementation with 4.8 g/day, aligning with findings in adults [59]. However, the absence of post-intervention muscle carnosine measurements limits the ability to confirm accumulation in this age group. While paresthesia is the most common side effect, it is transient and not considered harmful, though it may affect performance due to discomfort [104]. As such, β-alanine may be beneficial under medical supervision for elite adolescent athletes engaged in anaerobic sports. Creatine monohydrate is one of the most effective ergogenic supplements for enhancing high-intensity exercise performance by increasing intramuscular phosphocreatine stores and accelerating ATP regeneration. Studies in adolescents, although limited and primarily focused on swimmers and soccer players, have reported improvements in sprint ability, muscular strength, power output, and sport-specific skills, with no adverse effects observed. Loading protocols (20–30 g/day for 5–7 days) followed by maintenance doses (3–5 g/day) are commonly used, although long-term safety data in youth populations are still scarce [46,47,48,50,51]. According to the International Society of Sports Nutrition [87], creatine may be considered safe and beneficial for adolescent athletes under professional supervision, particularly in anaerobic or strength-based sports. Nonetheless, further research is required to establish long-term safety and efficacy in this age group. Other bioactive compounds, such as omega-3 fatty acids and antioxidants (e.g., vitamin C, vitamin E, polyphenols), are also being explored in youth athletes. Omega-3s may offer anti-inflammatory benefits and support recovery, while antioxidants could help counteract exercise-induced oxidative stress [67,68,69]. However, the efficacy and necessity of such supplements in healthy adolescents are still debated, and routine use is not currently recommended without specific deficiencies or medical indications.

Overall, the current body of evidence suggests that some ergogenic aids, such as caffeine and potentially β-alanine, may offer performance benefits to adolescent athletes when used appropriately. However, the limited number of high-quality, long-term studies in this population makes it difficult to generalize recommendations. Individualization of supplementation, consideration of sex differences, genetic variability, and a careful assessment of side effects are essential to ensure both efficacy and safety. Furthermore, ethical considerations around supplement use in youth athletes must not be overlooked, particularly in recreational settings where the risk-to-benefit ratio may not justify their use. Despite the growing interest in dietary supplements and bioactive compounds in young athletes, the current literature is limited in both quantity and quality, especially when compared to adult populations. This lack of pediatric-focused data poses a significant risk, as recommendations are often extrapolated from adult studies without proper validation in youth. When evidence is insufficient or contradictory, caution is needed to avoid inappropriate or potentially harmful practices. In this context, the role of a well-balanced, age-appropriate diet should always take priority over supplementation, particularly in growing individuals with unique physiological needs. Moreover, rather than focusing solely on isolated nutrients, future studies should consider interventions aimed at improving the overall dietary patterns of young athletes. These approaches may have greater long-term health and performance benefits, with fewer risks and side effects. We believe these elements are essential to guide practitioners, families, and coaches toward responsible, evidence-based nutritional strategies.

## 5. Conclusions

In conclusion, while the use of ergogenic supplements and other bioactive compounds, such as creatine, omega-3 fatty acids, vitamin C, vitamin E, and polyphenols, is becoming increasingly common among adolescent athletes, the scientific evidence supporting their efficacy and safety in this age group remains limited. Among the most studied substances, caffeine shows promising ergogenic effects even at low doses, particularly for anaerobic performance. In contrast, due to limited evidence in adolescents, the use of β-alanine cannot be currently recommended, and further research is needed to establish its safety and efficacy in this population. Further studies are also needed to better assess the long-term safety and efficacy of creatine supplementation in adolescent athletes. Conversely, dietary nitrates have not consistently demonstrated performance benefits in adolescent athletes, likely due to physiological and methodological factors that require further exploration. It is also important to emphasize that the use of general dietary supplements in adolescents, including multivitamins and immune boosters, should be based on individual needs rather than generalized trends and should not replace a well-balanced diet. In addition to traditional ergogenic acids, bioactive compounds such as curcumin and omega-3 fatty acids are gaining interest for their potential to modulate inflammation, oxidative stress, and recovery in athletes. Although some evidence in adult populations suggests benefits in terms of muscle soreness, immune function, and training adaptation, studies in adolescents are extremely limited. Given the differences in metabolism, developmental stage, and nutrient requirements, more research is needed to assess the role and safety of bioactive compounds in young athletes before routine use can be recommended.

Given the physiological differences between adolescents and adults, as well as the ethical and developmental considerations involved, the use of performance-enhancing supplements in youth should be approached with caution. Personalized assessment, professional guidance, and evidence-based protocols are essential to ensure both effectiveness and safety. Furthermore, the psychological implications of supplements such as dependency, body image concerns, and performance pressure must be carefully considered, particularly in competitive and recreational youth settings. Regulatory oversight, the education of families and coaches, and interdisciplinary collaboration are critical to safeguarding adolescent health and promoting informed choices. Future research should prioritize well-designed, long-term studies that include both male and female adolescent athletes, investigate sex and maturity specific responses, and evaluate not only performance but also health, psychological, and ethical outcomes. Only through a comprehensive and individualized approach can we ensure that nutritional strategies in youth sports truly support athletic development, well-being, and long-term health.

## Figures and Tables

**Table 1:** Classification of athletes according to McKinney et al. [9].

Athlete Category	Training Volume	Competition Level
Elite athletes	>10 h/week	Performances at the highest level of competition
Competitive athletes	>6 h/week	Emphasis on improving performance
Recreational athletes	>4 h/week	Unregulated competitions
Exercises	>2.5 h/week	Maintain health and fitness status

**Table 2:** Adapted nutritional strategies for adolescent athletes based on adult recommendations, including guidance on macronutrient distribution, essential micronutrient intake, and hydration strategies.

**Macronutrients**		
**Component**	**Recommendation**	**Details**
**Carbohydrates**	45–60% of total caloric intake [21]	Favoring low-glycemic sources (e.g., whole grains, fruits).
	8–12 g of carbohydrate/kg/day [g/kg/day] [22]	To optimize muscle glycogen stores for athletes completing high volumes (i.e., ≥8 h) of exercise per week.
	Pre-exercise: 1–4 g/kg consumed 1–4 h before exercise [18]	Before exercise > 60 min
	During exercise > 60 min duration: 30–60 g/h and up to 90 g/h for very prolonged events (2.5+ h) [18]	The benefits are related to muscle energy demands and the maintenance of blood glucose levels.
	Post-exercise: 1.2 g/kg/h for 4–6 h soon after exhausting exercise, with a preference towards carbohydrate sources that have a high (>70) glycemic index [22]	When rapid restoration of glycogen is required (<4 h of recovery time).
**Proteins**	1.2–2.0 g/kg body weight/day and regular spacing of intakes of modest amounts of high-quality protein throughout the day [18]	To support lean mass replenishments and growth.
	0.3 g/kg body weight high-quality protein after exercise [18]	To enhance muscle protein synthesis and exercise recovery.
	Higher protein intakes when energy availability is reduced (e.g., to reduce body weight/fat) [18]	They are needed to support muscle protein synthesis (MPS) and retention of fat-free mass.
**Fats**	20–35% of total energy intake [18]	Essential for absorption of fat-soluble vitamins and hormone production.
	Athletes should be discouraged from chronic implementation of fat intakes below 20% of energy intake	Reduction in dietary variety often associated with such restrictions is likely to reduce the intake of fat-soluble vitamins and essential fatty acids, especially n-3 fatty acids.
	Intake of fat by athletes should be in accordance with public health guidelines and should be individualized based on training level and body composition goals [18]	Follow public health guidelines while adjusting according to sport, training intensity, and body composition targets.
**Micronutrients** Athletes should have diets providing at least the Recommended Dietary Allowance (RDA) for all micronutrients. Vitamin and mineral supplements are unnecessary for the athlete who consumes a diet providing high-energy availability from a variety of nutrient-dense foods [18]. PRI refers to Population Reference Intake, AI to Adequate Intake, UL to Upper Level.		
**Components**	**Recommendation**	**Details**
**Iron** [21]	7–11 years 11 mg/day PRI (females and males) 12–17 years 13 mg/day PRI (females) and 11 mg/day PRI (males)	Heme and non-heme iron from food sources (meat, legumes, fortified cereals). Adolescent females require a higher intake to compensate for menstrual losses. Iron absorption is improved by vitamin C and reduced by tannins and phytates.
**Vitamin D** [21]	7–10 years 15 μg/day AI and 50 μg VDE/day UL (females and males) 11–17 years 15 μg/day AI and 100 μg VDE/day UL (females and males)	VDE: vitamin D equivalent
**Calcium** [21]	4–10 years 800 mg/day PRI (females and males) 11–17 years 1150 mg/day PRI (females and males)	Essential for bone mineralization and skeletal growth during childhood and adolescence. Main sources: dairy products, green leafy vegetables, calcium water, fortified foods.
**Hydration guidelines**		
**Aspect**	**Recommendation**	
**Fluid loss**	Avoid >2% body mass loss due to sweat [23]	Dehydration exceeding 2 percent of body weight impairs physical and cognitive performance. Monitor pre- and post-exercise body weight to estimate sweat losses.
**Fluid intake during high-intensity exercise (>60 min)**	Use sport drinks (probably better than water alone) [23]	Drinks with carbohydrates (4–8%) and electrolytes are preferable to water to maintain blood sugar levels, delay fatigue, and promote fluid absorption during prolonged or intense exercise. Strategies should be individualized according to sweating rate and environmental conditions.
**Fluid intake after exercise for restore balance**	Drink ~1.5 L of fluid for each kg of body weight lost during exercise [23]	For individuals requiring rapid and complete recovery from excessive dehydration.

**Table 3:** Reference age, dosage, and duration of administration of bioactive compounds in clinical trials and meta-analyses targeting the pediatric population of young athletes.

Bioactive Compound or Dietary Supplement	Author (Year of Publication), Study Design	Dosage Under Investigation	Duration of Administration	Reference Age	Outcome of Interest
*Micronutrients of key interest for prevention, well-being, and performance*					
Vitamin D	Bezrati I. et al. (2020); RCT [24]	200.000 UI	Acute administration	School-age children and adolescent males (8–15 years)	Physical performance
	Dubnov-Raz G. et al. (2015); RCT [25]	2000 UI/day	12 weeks	Adolescent males (12–18 years)	
Calcium	Azevedo H. et al. (2024); clinical trial [26]	21.5 mg/kg body mass	Acute administration (60 min before the tests)	Adolescence soccer players (<15 years)	Athletic performance
Iron	Gera T. et al. (2007); systematic review of randomized controlled trials [27]	30–200 mg/day	1–2 months	School-age children and adolescents (8–15 years) and adolescent girls (<18 years)	Physical performance
	Pasricha S.R. et al. (2014); systematic review of randomized controlled trials [28]	1–325 mg/day	4–24 weeks	12–50 years adolescent and adult women	
*Supplements that may assist with training capacity, recovery, muscle soreness, and injury*					
Omega-3	Guezennec C.Y. et al. (1989); clinical trial [29]	* no data in pediatric age 6 g/day (no information regarding EPA and DHA)	6 weeks	Adults (19–38 years)	Anti-inflammatory effects
	Brilla L.R. and Landerholm T.E. (1990); clinical trial [30]	* no data in pediatric age 4 g/day (no information regarding EPA and DHA)	10 weeks	Adults (19–34 years)	
	Raastad T. et al. (1997); RCT [31]	* no data in pediatric age 5.2 g/day (1.60 g/day EPA and 1.04 g/day DHA)	10 weeks	Adults (age ~20 yrs)	
	Smith G.I. et al. (2011); RCT [32]	* no data in pediatric age 4 g/day (1.86 g EPA and 1.5 g DHA)	8 weeks	Adults (25–45 yrs)	
	Smith G.I. et al. (2011); RCT [33]	* no data in pediatric age 4 g/day (1.86 g EPA and 1.5 g DHA)	8 weeks	Adults (≥65 yrs)	
Vitamin C	Schröder H. et al. (2001); clinical trial [34]	* no data in pediatric age Vitamin C (1000 mg/day), vitamin E (600 mg/day), β-carotene (32 mg/day)	35 day	Young adults (23–24 years)	Antioxidant effects and risk of upper respiratory infections
	Mastaloudis A. et al. (2006); RCT [35]	* no data in pediatric age Vitamin C (1000 mg/day), vitamin E (300 mg/day)	6 weeks; 2 h before the exercise test	39–41 years	
	Constantini N.W et al. (2011); RCT [36]	Vitamin C (500 mg/twice a day)	3 months	12–17 years	
	Ashton T. et al. (1999); clinical trial [37]	* no data in pediatric age Vitamin C (1000 mg/day)	(2 h before exercise) 10 days (during training camp)	18–30 years	
	Żychowska M. et al. (2015); clinical trial [38]	Vitamin C (8 mg/kg/day), vitamin A (16 μg/kg/day), vitamin E (1 mg/kg/day)	10 days (during training camp)	Adolescents (14–15 years)	
	Thompson D. et al. (2001); RCT [39]	* no data in pediatric age Vitamin C (400 mg/day)	14 days pre-exercise + 3 days post-exercise	Healthy adult males (~20–45 years)	
	Ristow M. et al. (2009); RCT [40]	* no data in pediatric age Vitamin C (1000 mg/day), vitamin E (400 UI/day)	4 weeks	Healthy adult males (25–35 y)	
Curcumin	Sahebkar A. et al. (2015); systematic review and meta-analysis of RCT [41]	* no data in pediatric age 80–1500 mg/day (different formulations)	≥6 weeks	Adults (age not specified)	Antioxidant and anti-inflammatory effects
	Daniel Vasile P.R. et al. (2024); systematic review [42]	* no data in pediatric age 90–5000 mg/day (different formulations)	Before and/or after exercise, up to 72 h post-exercise	Adult athletes (>18 years)	
	Heidari Z. et al. (2022); systematic review [43]	* no data in pediatric age 45–4000 mg/day (different formulations)	2–48 weeks	Pediatric patients (2–18 years)	
	Bai K.Y. Et al. (2022); clinical trial [44]	1200 mg/day (powder form)	12 weeks	Adolescent athletes (mean age 17 years)	
	Suhett L.G. et al. (2021); systematic review [45]	* no data in pediatric age 10–6000 mg/day (different formulations)	1 day–3 months (mostly 7–28 days)	Adolescents (17 years mean)	
*Ergogenic supplements*					
Creatine	Juhász I. et al. (2009); RCT [46]	20 g/day	5 days	Adolescents (14–19 years)	Increased phosphocreatine stores and ATP production
	Claudino J.G. et al. (2014); RCT [47]	20 g/day (1 week), then 5 g/day (6 weeks)	7 weeks	17–19 years	
	Dawson B. et al. (2002); clinical trial [48]	20 g/day (5 days), then 5 g/day (22 days)	4 weeks	16 years	
	Grindstaff P.D. et al. (1997); RCT [49]	21 g/day	9 days	Adolescents (age not specified)	
	Ostojic S.M. (2004); clinical trial [50]	30 g/day	7 days	16–17 years	
	Yáñez-Silva A. et al. (2017); clinical trial [51]	0.03 g/kg/day	14 days	17–18 years	
	Theodorou A.S. et al. (1999); RCT [52] 27	25 g/day (4 days) then 5 g/day (2 months)	4 days load + 2 months maintenance	Age not specified	
Caffeine	Loureiro L.M.R. et al. (2018); systematic review [53]	* no data in pediatric age (5–10.2 mg/kg body weight)	Up to 4 h post-exercise	Adults (age not specified)	Neuromuscular function-enhancing effects
	Ghazaleh L. et al. (2024); RCT [54]	6 mg/kg body weight	Administration 60 min pre test	Adolescents (14.7–17.5 years)	
	Jordan J.B. et al. (2014); RCT [55]	6 mg/kg body weight	30 min pre test	Adolescents (14 years)	
	Stojanović E. et al. (2022); RCT [56]	6 mg/kg body weight (double administration of 3 mg/kg body weight)	Administration 30–60 min pre test	Adolescents (15–16 years)	
	Ellis M. et al. (2019); RCT [57]	1, 2 or 3 mg/kg body weight	Administration 30–60 min pre test	Adolescents (16–17 years)	
Beetroot Juice	Wong T.H. et al. (2021); systematic review [58]	* no data in pediatric age 70–500 mL/day (from 4.84 mmol NO3-/day to 29 mmol NO3- in 36 h) and different formulations	Administration 60 min pre test	Adolescents (16–17 years)	nitric oxide (NO)-enhancing effect
Beta-alanine	Claus G.M. et al. (2017); RCT [59]	6.4 g/day	6 weeks	Adolescents (14–18 years)	Intracellular acid-buffering effect

* is to highlight the fact that they are articles on an adult population and not a paediatric one.

## Supplementary Materials

The following supporting information can be downloaded at: https://www.mdpi.com/article/10.3390/nu17132194/s1.
