# Supplementary figures and images for: Use of Nutritional Strategies, Bioactive Compounds, and Dietary Supplements in Young Athletes: From Evidence to Potential Risks—A Narrative Review
Source: Nutrients. 2025 Jun 30;17(13):2194. doi: 10.3390/nu17132194 (PMC12251387; doi:10.3390/nu17132194)

### Flowchart of study selection

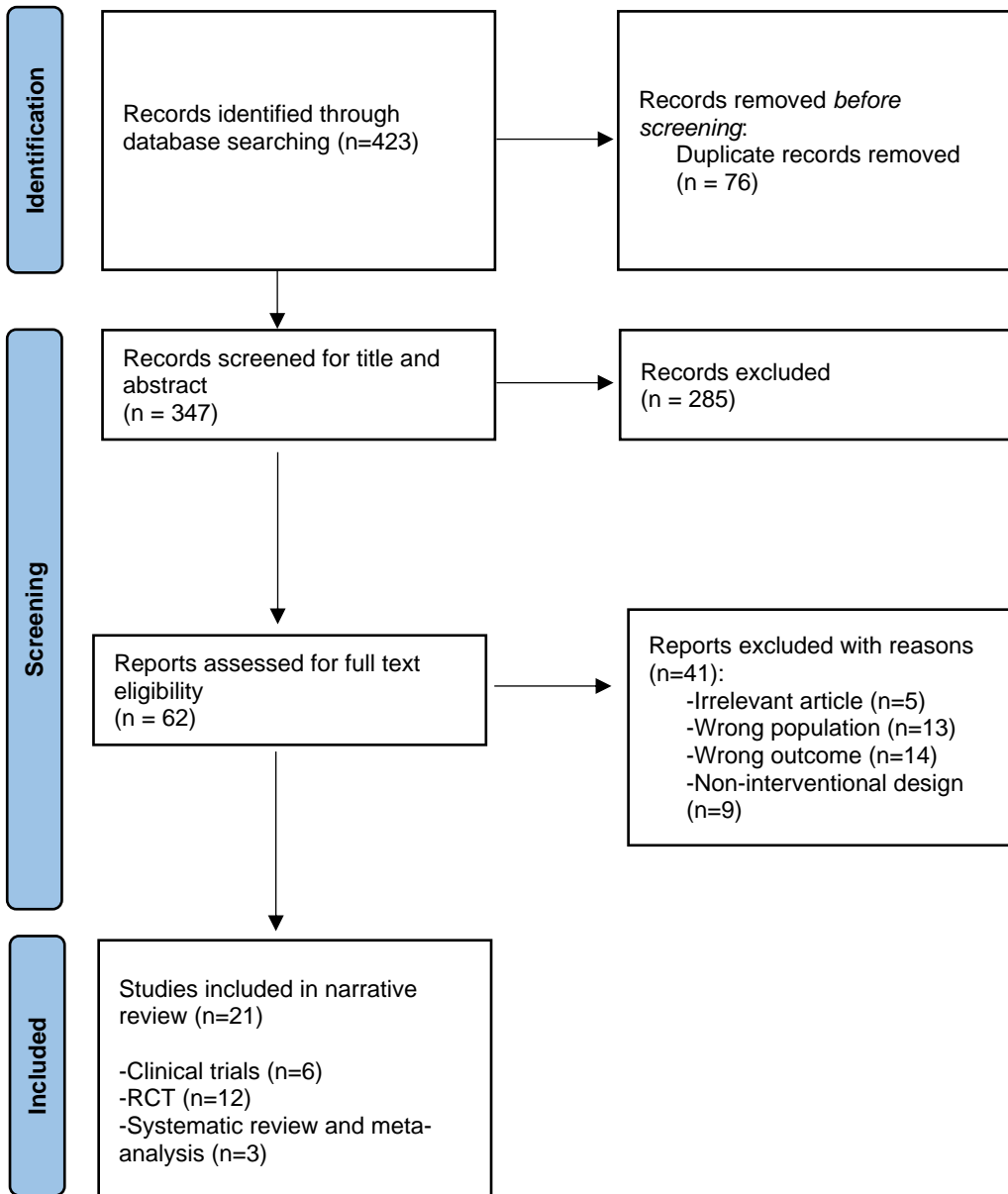

Supplement: Supplementary file 1 [file nutrients-17-02194-s001.zip › nutrients-3689113-supplementary.pdf]
